# Cerebroplacental ratio and middle cerebral artery doppler indices as associations with maternal and neonatal outcomes in term pregnancies undergoing labor induction or augmentation: A cross-sectional study

**DOI:** 10.1016/j.eurox.2026.100481

**Published:** 2026-07-31

**Authors:** Fatemeh Bahadori, Leila Majdi, Neda Rashidian, Peyman Moshari

**Affiliations:** a Department of Perinatology, Kosar Hospital, Urmia University of Medical Sciences, Urmia, Iran; b Preventative Gynecology Research Center, Shahid Beheshti University of Medical Sciences, Tehran, Iran; c Urmia University of Medical Sciences, Urmia, Iran

**Keywords:** Cerebroplacental ratio, Middle cerebral artery Doppler, Umbilical artery Doppler, Labor induction, Labor augmentation, Perinatal outcomes, Fetal surveillance, Term pregnancy

## Abstract

**Background:**

Placental insufficiency remains a leading cause of adverse perinatal outcomes. The cerebroplacental ratio (CPR) has emerged as a hemodynamic marker reflecting fetal adaptive responses to hypoxia. Its predictive value in term pregnancies requiring obstetric intervention to achieve delivery (labor induction or augmentation) requires further characterization.

**Objectives:**

Building on this background, our goal was to evaluate the association between middle cerebral artery (MCA) Doppler parameters and CPR with maternal and neonatal outcomes in term pregnant women undergoing labor induction or augmentation.

**Methods:**

This cross-sectional study enrolled 314 singleton term pregnancies (≥37 weeks) admitted for labor induction or augmentation at Kosar Hospital, Urmia, Iran (April 2022–January 2023). Participants underwent pre-induction Doppler ultrasonography.

We assessed MCA pulsatility index (PI), umbilical artery (UA) PI, and CPR (MCA-PI/UA-PI). Primary outcomes included mode of delivery, 5-minute Apgar score, umbilical cord arterial pH, and neonatal intensive care unit (NICU) admission. We employed Chi-square tests, multivariable logistic regression, and receiver operating characteristic (ROC) curve analysis.

**Results:**

Mean maternal age was 30.01 ± 7.42 years with gestational age of 39.29 ± 1.02 weeks. Cesarean delivery occurred in 116 cases (36.9%). Abnormal CPR (<1.08) was identified in 51 participants (16.2%). Abnormal CPR was significantly associated with higher cesarean rates (72.5% vs. 30.0%; adjusted odds ratio [OR]=6.12, 95% confidence interval [CI]: 3.18–11.78), increased NICU admission (9.8% vs. 1.1%; P = 0.001), and reduced cord arterial pH (7.17 ± 0.06 vs. 7.20 ± 0.05; P = 0.007**).** However, sensitivity analysis restricted to objective cesarean indications (failure to progress and failed induction) showed attenuation of this association (OR=4.56; 95% CI: 2.34–8.89), suggesting the primary cesarean association may be partially influenced by clinician knowledge of CPR status. CPR demonstrated moderate discriminative ability compared to individual Doppler indices (area under the curve for cesarean delivery: 0.71; 95% CI: 0.64–0.78).

**Conclusions:**

Low CPR in term pregnancies undergoing labor induction or augmentation shows a significant association with adverse perinatal outcomes. However, the association with cesarean delivery should be interpreted cautiously given the lack of clinician blinding, which may have substantially inflated this relationship. CPR demonstrates moderate discriminative ability for objective neonatal outcomes (cord pH, NICU admission), which are less susceptible to observer bias. The moderate area under the curve (0.71–0.78) and low sensitivity (32–63%) indicate that CPR has limited value as a standalone screening test, and many fetuses destined for adverse outcomes will have normal CPR values. CPR should not be used to guide clinical decision-making in isolation. Well-designed prospective trials with clinician blinding are essential to establish whether CPR-guided management improves perinatal outcomes and to quantify the true predictive value of this marker independent of observer bias.

## 1 Introduction

Optimal fetal growth depends on a complex interplay between genetic potential and environmental factors, mediated by maternal, fetal, and placental contributions. When this developmental trajectory becomes compromised, fetal growth restriction (FGR) occurs, affecting approximately 5–10% of all pregnancies and conferring substantial morbidity [1], [2]. Uteroplacental insufficiency—encompassing vascular maldevelopment, impaired spiral artery remodeling, and disrupted feto-maternal exchange—represents the predominant underlying mechanism [3].

Clinicians commonly rely on the small-for-gestational-age (SGA) designation as a surrogate marker for FGR. SGA is defined as estimated fetal weight or birth weight below the 10th percentile [4]. This approach has inherent limitations, as SGA encompasses constitutionally small but healthy fetuses alongside those with genuine growth restriction. Consequently, size criteria alone demonstrate suboptimal predictive capacity for adverse outcomes, particularly in term gestations where traditional markers may appear reassuring despite underlying compromise [5], [6]. Universal third-trimester ultrasonography has shown promise in identifying such cases [7].

Early-onset and late-onset FGR differ in their pathophysiology, carrying important clinical implications. Early-onset FGR typically manifests before 32 weeks' gestation, resulting from profound placental maldevelopment and exhibiting characteristic Doppler abnormalities, including elevated umbilical artery resistance and abnormal venous waveforms [8]. In contrast, late-onset FGR presents a more subtle phenotype. It often evades detection through conventional surveillance methods. In these cases, placental reserve may be sufficient under basal conditions. However, it may be inadequate to meet the escalating metabolic demands of advancing gestation [4]. Recent evidence demonstrates that combining computerized fetal heart rate analysis with ductus venosus Doppler improves outcome prediction in early-onset cases [9].

When faced with hypoxemia, the fetus redistributes cardiac output toward essential organs, with the brain receiving priority. This phenomenon, termed "brain-sparing" [10], represents a compensatory mechanism that manifests as decreased cerebrovascular resistance in the MCA. It may be accompanied by increased placental vascular resistance, producing characteristic alterations in Doppler indices. The CPR integrates these hemodynamic changes into a single parameter, calculated as the ratio of the MCA pulsatility index to the umbilical artery pulsatility index. Notably, CPR may be more sensitive than either component alone [11]. Recent prospective studies have confirmed the diagnostic accuracy of Doppler ultrasound in predicting perinatal outcomes at term [12], [13], further emphasizing its value in clinical practice.

Investigators have extensively studied the clinical utility of CPR, with heterogeneous results. Some studies demonstrate robust associations between low CPR and adverse outcomes, including cesarean delivery for fetal distress, low Apgar scores, neonatal acidemia, and NICU admission [14], [15], [16]. Other studies report modest predictive value, particularly in low-risk populations [17], [18]. These discrepancies may reflect differences in study populations, gestational age at assessment, cut-off values, and outcome definitions. Notably, birthweight may independently influence the relationship between CPR and adverse outcomes [19]. A recent systematic review emphasized the need for standardized protocols and cut-off values in future studies [20].

Labor induction and augmentation present a unique clinical scenario warranting dedicated investigation**.** Induced or augmented labor subjects the fetus to uterine contractions, which transiently compromise uteroplacental perfusion. Fetuses with marginal placental reserve may tolerate this stress poorly, potentially manifesting intrapartum compromise that could be anticipated through pre-labor hemodynamic assessment [21], [22]. Despite this biological rationale, evidence specifically addressing the predictive value of Doppler indices in the pre-induction or augmentation setting remains limited.

The optimal CPR threshold for identifying at-risk fetuses remains contentious. Various cut-offs have been proposed, including the 5th or 10th percentile for gestational age and fixed values such as 1.0 or 1.08 [16], [23]. The threshold of 1.08 corresponds approximately to the 5th percentile at term and has been validated in several populations [15], [24].

We designed this study to evaluate the association between MCA Doppler parameters and CPR with maternal and neonatal outcomes in pregnant women at term undergoing labor induction or augmentation. We hypothesized that abnormal CPR would demonstrate significant associations with adverse perinatal outcomes. We further hypothesized that CPR would demonstrate stronger associations across multiple outcome domains compared to individual Doppler indices.

## 2 Materials and methods

### 2.1 Study design and reporting standards

We conducted this cross-sectional analytical study at Kosar Hospital, Urmia, Iran. Kosar Hospital is a tertiary referral center affiliated with Urmia University of Medical Sciences. The study period extended from April 2022 to January 2023. This study adheres to the Strengthening the Reporting of Observational Studies in Epidemiology (STROBE) guidelines for reporting cross-sectional studies [25]. The completed STROBE checklist is provided as Supplementary File S4.

The Institutional Ethics Committee of Urmia University of Medical Sciences approved the study protocol (Ethics Code: IR.UMSU.REC.1401.159). All procedures adhered to the Declaration of Helsinki. All participants provided written informed consent before enrollment. Participation was entirely voluntary.

### 2.2 Study population

We employed consecutive sampling to enroll pregnant women admitted for labor induction or augmentation during the study period. We distinguished between these two clinical scenarios. Labor induction refers to artificial initiation of uterine contractions in women not in spontaneous labor. Labor augmentation refers to the enhancement of contractions in women who have already begun spontaneous labor but require assistance to progress.

Rationale for combined analysis: We analyzed both groups together in the primary analysis for several reasons. First, both groups share the common exposure of interest: pre-delivery Doppler assessment performed before active labor stress. Second, both groups face similar intrapartum hemodynamic stresses once active labor is established, regardless of how labor was initiated. Third, the clinical question of interest—whether CPR is associated with adverse outcomes in women requiring obstetric intervention to achieve delivery—applies to both populations. Fourth, combining groups maximizes statistical power for detecting clinically meaningful associations. However, we performed pre-specified subgroup analyses to assess whether CPR associations differed between these groups (see 3.9), and found no significant interaction (P = 0.82), supporting the validity of the combined analysis.

Inclusion criteria: (1) singleton pregnancy; (2) gestational age ≥ 37 completed weeks confirmed by first-trimester ultrasound or reliable last menstrual period; (3) planned labor induction or augmentation; (4) intact membranes at enrollment; and (5) willingness to provide informed consent.

Exclusion criteria: (1) multiple gestation; (2) known major fetal anomalies; (3) previously diagnosed intrauterine growth restriction with ongoing specialized surveillance; (4) non-cephalic presentation; (5) contraindications to vaginal delivery; (6) incomplete Doppler assessment; and (7) withdrawal of consent.

### 2.3 Sample size determination

We calculated the sample size based on the primary objective of detecting a significant difference in cesarean delivery rates between normal and abnormal CPR groups. We assumed a cesarean rate of 25% in the normal CPR group and 50% in the abnormal CPR group. We assumed abnormal CPR prevalence of 15%, α= 0.05 (two-tailed), and power= 80%. Sample size was calculated using G*Power software version 3.1.9.7 (Heinrich-Heine-Universität Düsseldorf, Germany) [26]. We determined that a minimum of 280 participants was required. We aimed to enroll approximately 320 participants to account for potential exclusions.

### 2.4 Data collection and doppler assessment

All participants underwent a comprehensive ultrasonographic evaluation before initiation of cervical ripening or oxytocin administration. A single experienced maternal-fetal medicine specialist performed all examinations. We used a Voluson E8 ultrasound system (GE Healthcare, Austria) equipped with a 2–5 MHz convex transducer. The sonographer had no knowledge of subsequent delivery outcomes at the time of assessment. We performed fetal biometry according to standardized protocols. We calculated the estimated fetal weight using the Hadlock formula. This formula incorporates biparietal diameter, head circumference, abdominal circumference, and femur length.We performed Doppler velocimetry during periods of fetal quiescence and apnea:

Umbilical Artery (UA) Doppler: We identified the free-floating loop of the umbilical cord. We applied pulsed-wave Doppler with an angle of insonation < 30°. We recorded three consecutive uniform waveforms. We calculated the mean pulsatility index (PI) and resistive index (RI).

Middle Cerebral Artery (MCA) Doppler: We imaged the fetal head in an axial plane at the level of the thalami and cavum septum pellucidum. Using color flow mapping, we identified the circle of Willis. We sampled the MCA in the proximal third, immediately after its origin from the internal carotid artery. We recorded peak systolic velocity (PSV), PI, and RI from uniform waveforms. We obtained these with an angle of insonation as close to 0° as possible.

Cerebroplacental Ratio (CPR): We calculated CPR as MCA-PI divided by UA-PI. We considered a CPR value < 1.08 abnormal based on established literature thresholds [15], [24]. This pre-specified threshold was selected before data analysis based on prior validation studies. We selected a fixed threshold rather than gestational age-specific centiles for several reasons. First, recent evidence suggests that CPR values remain relatively stable across term gestations (37–41 weeks) [15], [24], reducing the necessity for gestational age adjustment in this narrow window. Second, fixed thresholds offer practical advantages in clinical implementation, avoiding the need for reference charts and percentile calculations. Third, the threshold of 1.08 corresponds approximately to the 5th percentile at term and has been validated across multiple populations [15], [24]. Fourth, our data confirmed stability of CPR across gestational weeks (Table 5; Kruskal-Wallis P = 0.421), supporting the appropriateness of a fixed cut-off in this population. However, we acknowledge that gestational age-specific centiles may offer improved performance in broader gestational age ranges or when comparing preterm versus term fetuses.

The median time interval from Doppler assessment to delivery was 8.2 h (interquartile range: 4.5–14.6 h). All ROC analyses were independently verified by two authors (PM and NR). Figure preparation was cross-checked against statistical output to ensure concordance between graphical display and reported values.

### 2.5 Labor management and outcome assessment

Labor induction followed institutional protocols. We employed cervical ripening with prostaglandin E2 (dinoprostone) vaginal insert or misoprostol. Oxytocin infusion followed when indicated. We maintained continuous electronic fetal heart rate monitoring throughout labor.

Important methodological consideration regarding blinding: Clinicians managing labor were not blinded to Doppler results. Doppler findings were documented in the patient chart and were available to the managing obstetric team. This reflects real-world clinical practice where Doppler findings inform clinical decision-making. However, there was no formal protocol dictating specific management changes based on CPR values—clinicians retained full autonomy in labor management decisions.

Despite this autonomy, the lack of blinding represents a significant potential source of bias, particularly for the cesarean delivery outcome. Clinicians aware of abnormal CPR values may have had a lower threshold for proceeding to cesarean delivery when faced with ambiguous fetal heart rate patterns, which could inflate the observed association between CPR and cesarean delivery. This is especially relevant given that 44.8% of cesareans were performed for non-reassuring fetal heart rate patterns—a subjective assessment.

We address this limitation extensively in the Discussion (4.5 through sensitivity analysis restricting to objective cesarean indications (3.10). Interpretation of the cesarean delivery outcome should therefore be made with caution, and prospective blinded studies are needed to establish true predictive value.

Primary outcomes:

• Mode of delivery (vaginal vs. cesarean section)
• Indication for cesarean delivery
• Intrapartum fetal distress (non-reassuring fetal heart rate patterns necessitating intervention)
• Secondary outcomes:
• Birth weight and SGA status (birth weight <10th percentile using local standards)
• 5-minute Apgar score (categorized as <7 or ≥7)
• Umbilical cord arterial blood gas analysis (pH and base excess)
• Neonatal resuscitation requirement (positive pressure ventilation or greater intervention)
• NICU admission
• Neonatal mortality.

Composite adverse neonatal outcome: We defined this as the occurrence of any of the following: 5-minute Apgar score < 7, NICU admission, or neonatal resuscitation requirement. This composite endpoint increases statistical power for detecting associations with rare individual outcomes.

We obtained umbilical cord arterial blood samples immediately following delivery. We used a standardized double-clamp technique. We analyzed samples within 30 min using a point-of-care blood gas analyzer (ABL800 FLEX, Radiometer, Denmark). To ensure accurate arterial sampling, we compared arterial and venous pH values for each pair; samples were excluded if the arterio-venous pH difference was < 0.02, suggesting venous contamination or sampling error. The median time from cord clamping to blood gas analysis was 8.5 min (IQR: 5–15 min). All samples were maintained at room temperature and analyzed within 30 min as per institutional protocol.

Bishop score was not systematically recorded at the time of Doppler assessment in our study protocol, representing a limitation. Cervical favorability is a strong independent predictor of successful induction and may confound the association between CPR and cesarean delivery. We agree that failing to record the Bishop score could lead to residual confounding in the relationship between CPR and cesarean delivery.

### 2.6 Statistical analysis

We analyzed data using IBM SPSS Statistics version 26.0 (IBM Corporation, Armonk, NY, USA). We assessed continuous variables for normality using the Kolmogorov-Smirnov test. We expressed normally distributed variables as mean ± standard deviation (SD). We reported categorical variables as frequencies and percentages.

For group comparisons (normal vs. abnormal CPR), we employed an independent samples *t*-test or a Mann-Whitney *U* test for continuous variables. We used the chi-square test or Fisher's exact test for categorical variables. We assessed correlations using Spearman's rank correlation coefficient.

We performed multivariable logistic regression to assess independent associations between CPR status and primary outcomes. We adjusted for maternal age, parity, gestational age, and labor onset type. We assessed model fit using the Hosmer-Lemeshow goodness-of-fit test. We evaluated multicollinearity using variance inflation factors (VIF <5 considered acceptable).

We performed ROC curve analysis to evaluate the discriminative ability of CPR for predicting adverse outcomes. We calculated the area under the curve (AUC) with 95% CIs. We evaluated diagnostic performance at our pre-specified cut-off of 1.08. We calculated the Youden index (*J* = sensitivity + specificity − 1) for each outcome at this threshold. We considered two-tailed P-values < 0.05 statistically significant.

### 2.7 Ethical considerations

The Ethics Committee of Urmia University of Medical Sciences approved this study (Ethics Code: IR.UMSU.REC.1401.159). All participants provided written informed consent. We maintained patient confidentiality through data anonymization. We restricted database access to principal investigators.

## 3 Results

### 3.1 Study flow and baseline characteristics

**Fig. 1:**
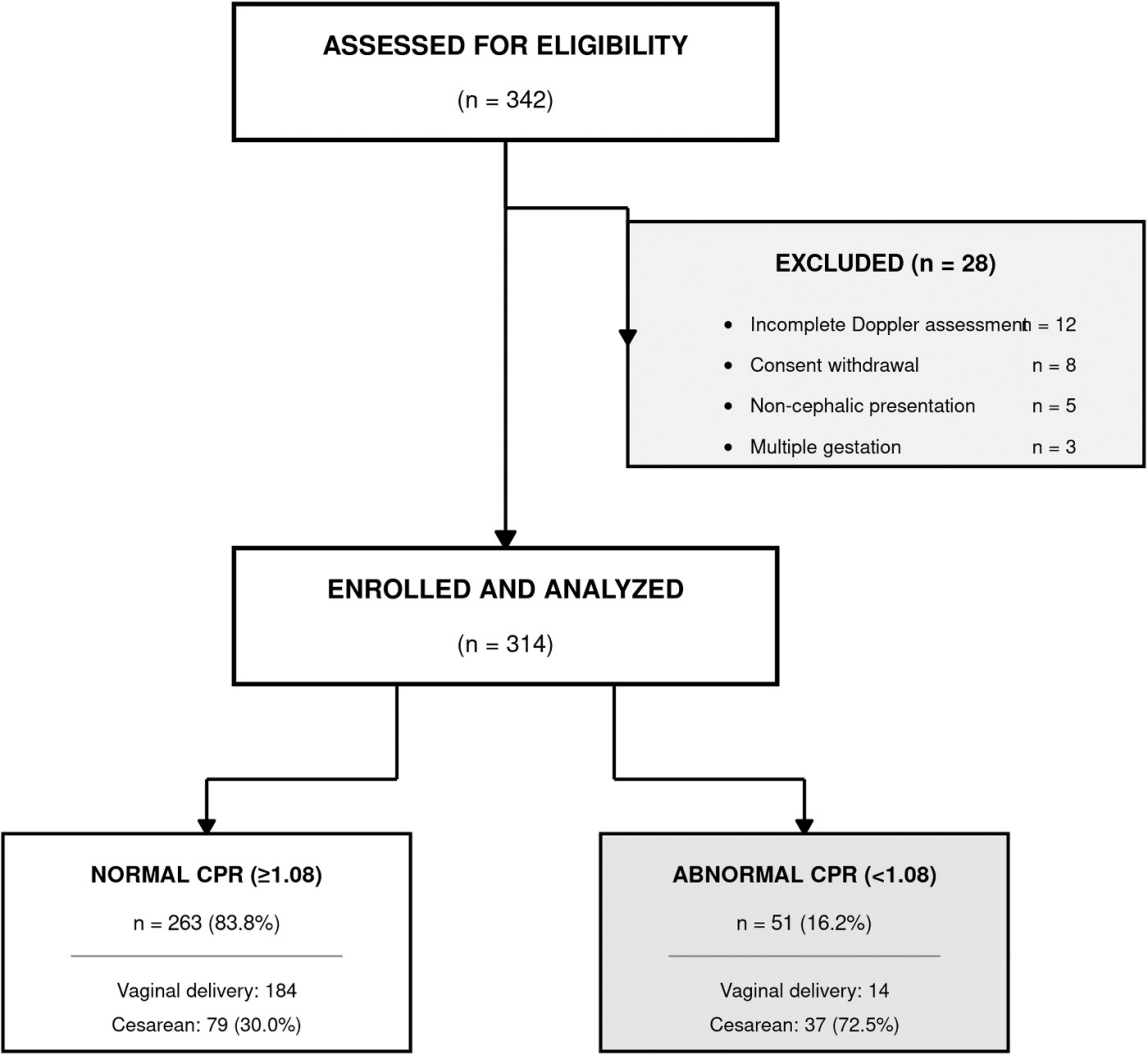
Study flow diagram showing participant recruitment and exclusions, Study flow diagram illustrating participant recruitment, exclusion criteria, and final study population stratified by cerebroplacental ratio (CPR) status. Of 342 women assessed for eligibility, 314 met inclusion criteria and were analyzed. Using a pre-specified CPR threshold of 1.08, participants were classified as having normal (n = 263, 83.8%) or abnormal (n = 51, 16.2%) CPR values.

During the study period, we assessed 342 women for eligibility (Fig. 1). We excluded 28: incomplete Doppler assessment (n = 12), consent withdrawal (n = 8), non-cephalic presentation discovered at admission (n = 5), and multiple gestation (n = 3). We included 314 participants in the final analysis.

Mean maternal age was 30.01 ± 7.42 years (range: 15–44 years). Gravidity ranged from 1 to 8. Primigravidae comprised the largest subgroup (n = 110; 35.0%). The parity distribution showed 126 nulliparous women (40.1%) and 188 multiparous women (59.9%). Table 1 presents the complete gravidity distribution. Supplementary Table S1 provides additional baseline characteristics stratified by CPR status.

**Table 1:** Distribution of Gravidity Among Study Participants.

**Gravidity**	**Frequency (n)**	**Percentage (%)**
1 (Primigravida)	110	35.0
2	76	24.2
3	58	18.5
4	42	13.4
5	16	5.1
6	4	1.3
7 +	8	2.5
Total	314	100.0

Mean gestational age at delivery was 39.29 ± 1.02 weeks (range: 37–41 weeks). The distribution by gestational week was: 37 weeks (n = 24; 7.6%), 38 weeks (n = 62; 19.7%), 39 weeks (n = 98; 31.2%), 40 weeks (n = 112; 35.7%), and 41 weeks (n = 18; 5.7%).

### 3.2 Labor onset and indications

3.2

Among the 314 participants, 190 (60.5%) presented with spontaneous labor onset requiring augmentation. The remaining 124 (39.5%) underwent elective labor induction. This distinction carries clinical significance. Women requiring augmentation had already demonstrated the capacity to initiate labor spontaneously. Those undergoing induction had not. We performed subgroup analyses to assess whether CPR associations differed between these groups (3.9).

Table 2 presents the complete indication distribution. Among those with specific induction indications, post-term pregnancy was most prevalent (n = 43; 13.7%). Suspected fetal compromise followed (n = 34; 10.8%).

**Table 2:** Indications for Labor Induction/Augmentation.

**Indication**	**Frequency (n)**	**Percentage (%)**
Spontaneous labor onset (augmentation)	190	60.5
Post-term pregnancy	43	13.7
Suspected fetal compromise	34	10.8
Preeclampsia	8	2.5
Suspected IUGR	8	2.5
Oligohydramnios	7	2.2
Prolonged labor	6	1.9
Maternal request (post-dates)	5	1.6
Intrahepatic cholestasis of pregnancy	5	1.6
Positive contraction stress test	4	1.3
Placental abruption (mild)	4	1.3
Total	314	100.0

IUGR: Intrauterine Growth Restriction.

### 3.3 Delivery outcomes

Vaginal delivery occurred in 198 women (63.1%). Cesarean section was performed in 116 cases (36.9%). Among cesarean deliveries, indications included: non-reassuring fetal heart rate tracing (n = 52; 44.8%), failure to progress (n = 38; 32.8%), failed induction (n = 18; 15.5%), and other indications (n = 8; 6.9%).

Mean birth weight was 3180.92 ± 510.36 g. SGA neonates numbered 39 (12.4%). NICU admission occurred in 8 neonates (2.5%).

The majority of neonates demonstrated favorable adaptation. A total of 306 (97.5%) achieved 5-minute Apgar scores ≥ 7. Table 3 presents the Apgar score distribution.

**Table 3:** Distribution of 5-Minute Apgar Scores.

**Apgar Score**	**Frequency (n)**	**Percentage (%)**
10	278	88.5
9	6	1.9
8	6	1.9
7	16	5.1
6	5	1.6
5	3	1.0
Total	314	100.0

Mean umbilical cord arterial pH was 7.199 ± 0.058 (range: 7.01–7.35). The mean arterial pH of 7.199 is at the lower boundary of the normal range (7.20–7.35) but remains within accepted limits. The median pH was 7.21 (IQR: 7.17–7.24). Only 12 neonates (3.8%) had arterial pH < 7.10, and none had pH < 7.00. The distribution reflects our study population, which included women with indications suggesting potential fetal compromise (suspected IUGR, oligohydramnios, post-term pregnancy), and the stress of induced/augmented labor. Mean base excess was −6.19 ± 0.81 mmol/L (range: −8.75 to −3.79). Table 4 presents these parameters.

**Table 4:** Umbilical Cord Arterial Blood Gas Parameters.

**Parameter**	**Minimum**	**Maximum**	**Mean ± SD**
pH	7.01	7.35	7.199 ± 0.058
Base Excess (mmol/L)	−8.75	−3.79	−6.19 ± 0.81

Nine neonates (2.9%) required resuscitation at birth. No neonatal or maternal deaths occurred.

### 3.4 Doppler velocimetry parameters

Mean Doppler parameters for the entire cohort were: MCA-PI 1.24 ± 0.42, MCA-RI 0.68 ± 0.11, UA-PI 0.83 ± 0.36, UA-RI 0.54 ± 0.13, MCA-PSV 46.07 ± 16.09 cm/s, and CPR 1.62 ± 0.74. Detailed distributions by gestational week are presented in Supplementary Table S5.

Doppler parameters remained stable across gestational weeks with two exceptions: UA-RI showed a statistically significant increase at 41 weeks compared to 40 weeks (0.67 vs 0.51; post-hoc P = 0.016), though the clinical significance of this finding is uncertain given the small sample at 41 weeks (n = 18).

Using the pre-specified cut-off of 1.08, we identified 51 participants (16.2%) with abnormal CPR (Table 5). Fig. 4 illustrates the CPR distribution across the study population. Supplementary Table S3 presents the correlation matrix for all Doppler parameters.

**Table 5:** Distribution of CPR Status.

**CPR Status**	**Frequency (n)**	**Percentage (%)**
Normal (≥1.08)	263	83.8
Abnormal (<1.08)	51	16.2
Total	314	100.0

### 3.5 Association between CPR and perinatal outcomes

Comparative analysis between normal and abnormal CPR groups revealed significant associations with multiple outcome variables. Table 6 presents these comparisons.

**Table 6:** Comparison of Outcomes by CPR Status.

**Variable**	**Normal CPR (n = 263)**	**Abnormal CPR (n = 51)**	**Test Statistic**	**P-value**
Continuous variables, mean ± SD				
Maternal age (years)	30.30 ± 7.37	28.60 ± 7.74	t = 1.59	0.113
Gestational age (weeks)	39.31 ± 1.00	39.18 ± 1.12	t = 0.97	0.334
Birth weight (grams)	3225.73 ± 468.12	3094.11 ± 667.68	t = 2.36	0.019
Cord arterial pH	7.20 ± 0.05	7.17 ± 0.06	t = 2.71	0.007
Base excess (mmol/L)	−6.11 ± 0.84	−6.62 ± 0.71	t = 4.12	< 0.001
Categorical variables, n (%)				
SGA (<10th percentile)	19 (7.2%)	20 (39.2%)	χ²= 36.8	< 0.001
Cesarean delivery	79 (30.0%)	37 (72.5%)	χ²= 31.4	< 0.001
NICU admission	3 (1.1%)	5 (9.8%)	χ²= 11.2	0.001
Resuscitation required	4 (1.5%)	5 (9.8%)	χ²= 9.1	0.003
Apgar < 7 at 5 min	3 (1.1%)	5 (9.8%)	χ²= 11.2	0.001
Composite adverse outcome	6 (2.3%)	9 (17.6%)	χ²= 19.8	< 0.001

SGA: Small for gestational age; NICU: Neonatal intensive care unit Bold P-values indicate statistical significance (P < 0.05).

### 3.6 Multivariable analysis

3.6

Multivariable logistic regression confirmed independent associations between abnormal CPR and adverse outcomes. We adjusted for potential confounders. Table 7 presents these results. The Hosmer-Lemeshow test indicated adequate model fit for all outcomes (P > 0.05). VIFs were < 2.0 for all covariates. Wide confidence intervals for NICU admission (95% CI: 2.11–43.12) reflect the small number of events (n = 8) and indicate substantial uncertainty in the point estimate. Fig. 3 presents the forest plot of adjusted ORs.

**Table 7:** Multivariable Logistic Regression for Primary Outcomes.

**Outcome Variable**	**Adjusted OR**	**95% CI**	**P-value**
Cesarean delivery			
Abnormal CPR (<1.08)	6.12	3.18–11.78	< 0.001
Nulliparity	2.34	1.45–3.78	< 0.001
Maternal age (per year)	1.02	0.98–1.06	0.284
Labor onset (induced vs. spontaneous)	1.48	0.91–2.42	0.116
Model: Hosmer-Lemeshow χ²= 6.24, P = 0.621; C-statistic= 0.74			
NICU admission			
Abnormal CPR (<1.08)	9.54	2.11–43.12	0.003
Nulliparity	1.82	0.41–8.06	0.432
Gestational age (per week)	0.71	0.38–1.32	0.281
Model: Hosmer-Lemeshow χ²= 4.18, P = 0.758; C-statistic= 0.81			
Composite adverse neonatal outcome			
Abnormal CPR (<1.08)	7.28	2.68–19.78	< 0.001
Nulliparity	1.56	0.58–4.19	0.378
SGA	3.12	1.08–9.01	0.036
Model: Hosmer-Lemeshow χ²= 5.82, P = 0.561; C-statistic= 0.79			

OR: Odds ratio; CI: Confidence interval; SGA: Small for gestational age.

### 3.7 ROC curve analysis

**Fig. 2:**
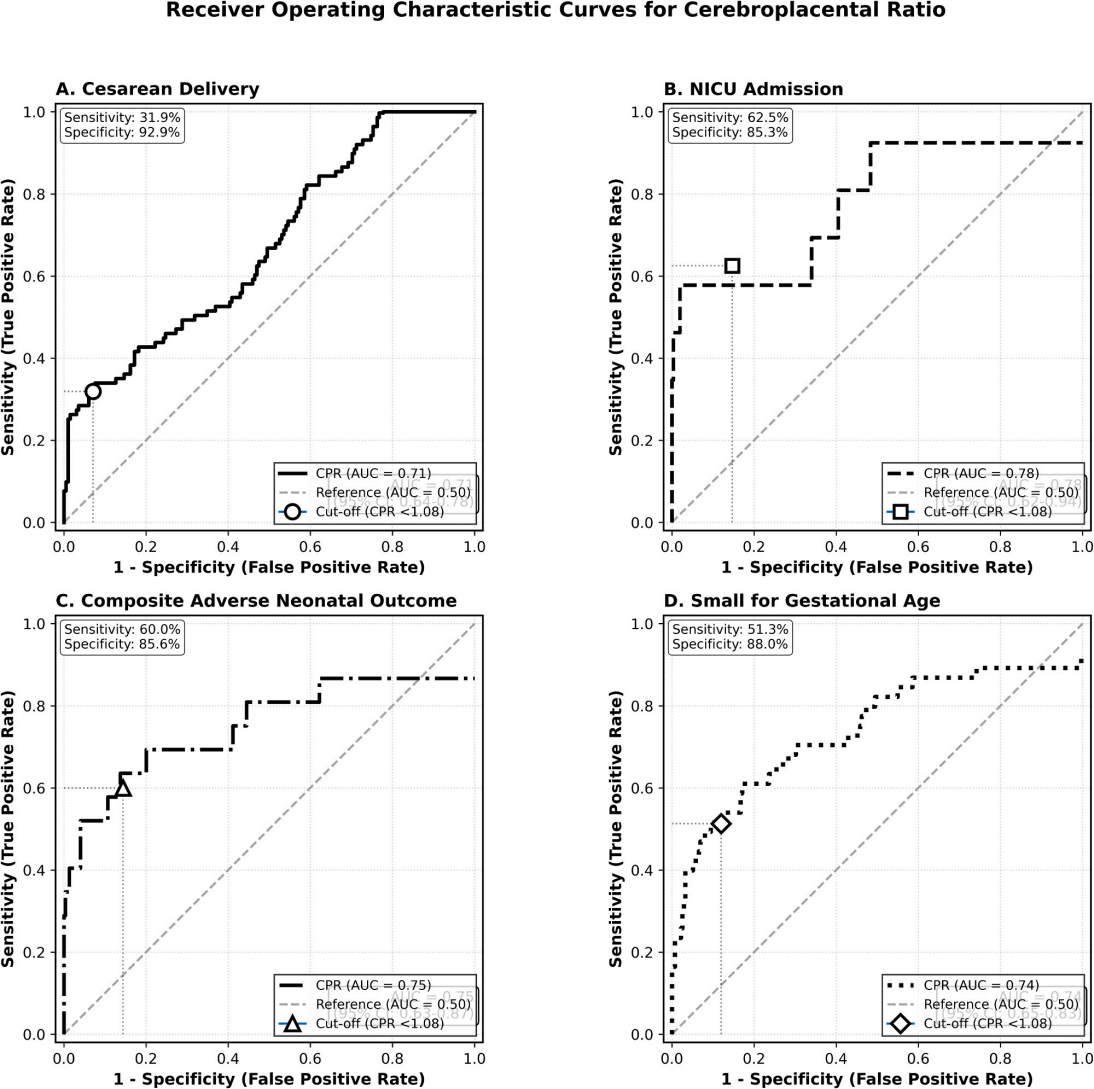
ROC Curves (4-Panel). Receiver operating characteristic (ROC) curves for cerebroplacental ratio predicting: (A) cesarean delivery, (B) NICU admission, (C) composite adverse neonatal outcome, (D) small-for-gestational-age status, Receiver operating characteristic (ROC) curves for cerebroplacental ratio (CPR) predicting adverse perinatal outcomes. (A) Cesarean delivery, area under the curve (AUC) = 0.71 (95% CI: 0.64–0.78); (B) Neonatal intensive care unit (NICU) admission, AUC = 0.78 (95% CI: 0.62–0.94); (C) Composite adverse neonatal outcome, AUC = 0.75 (95% CI: 0.63–0.87); (D) Small for gestational age (SGA), AUC = 0.74 (95% CI: 0.65–0.83). The diagonal dashed line represents the reference line (AUC = 0.50). The pre-specified cut-off of CPR < 1.08 is indicated on each curve.

**Fig. 3:**
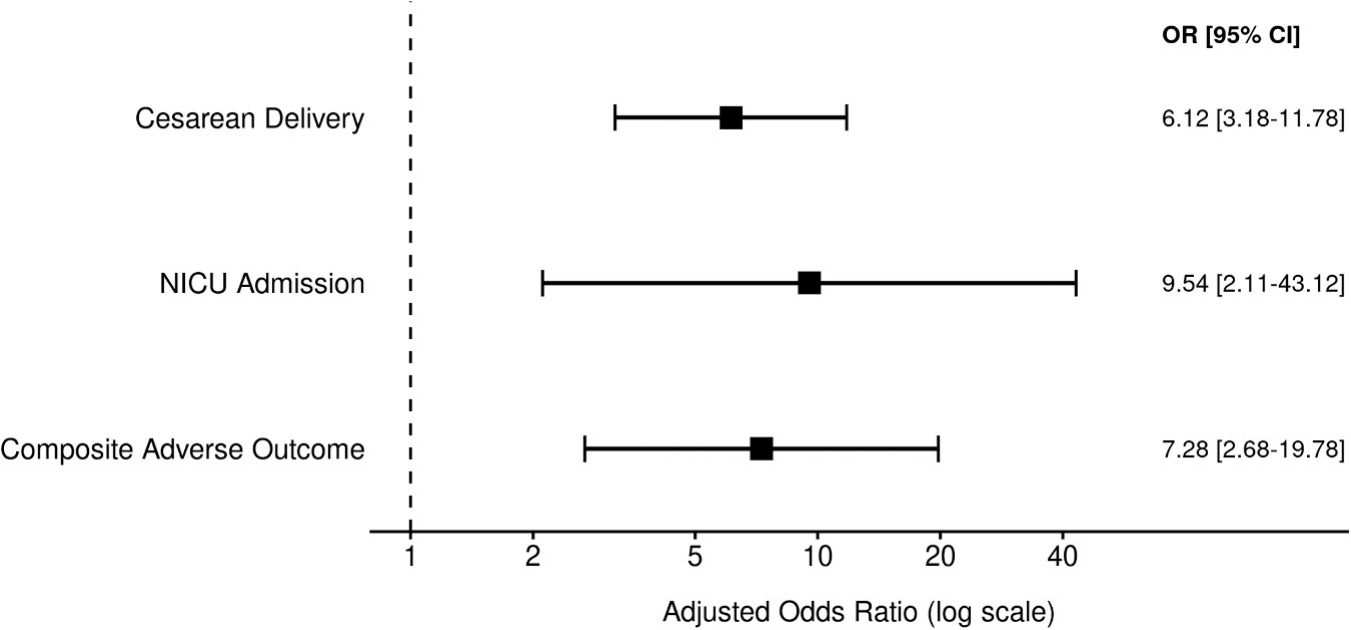
Forest plot showing adjusted odds ratios, Forest plot displaying adjusted odds ratios (ORs) with 95% confidence intervals (CIs) for adverse perinatal outcomes associated with abnormal cerebroplacental ratio (CPR <1.08) compared to normal CPR (≥1.08) in term pregnancies undergoing labor induction or augmentation (n = 314). ORs were adjusted for maternal age, parity, gestational age, and labor onset type (induction vs. augmentation). The vertical dashed line at OR= 1.0 represents the null hypothesis of no association. Points to the right of this line indicate increased odds of adverse outcomes with abnormal CPR. Primary outcomes shown include: cesarean delivery (adjusted OR=6.12; 95% CI: 3.18–11.78), though this may be partially inflated by lack of clinician blinding; NICU admission (adjusted OR=9.54; 95% CI: 2.11–43.12), with wide CI reflecting small event number; and composite adverse neonatal outcome (adjusted OR=7.28; 95% CI: 2.68–19.78). All associations achieved statistical significance (P < 0.05). Model fit was adequate for all outcomes (Hosmer-Lemeshow P > 0.05).

**Fig. 4:**
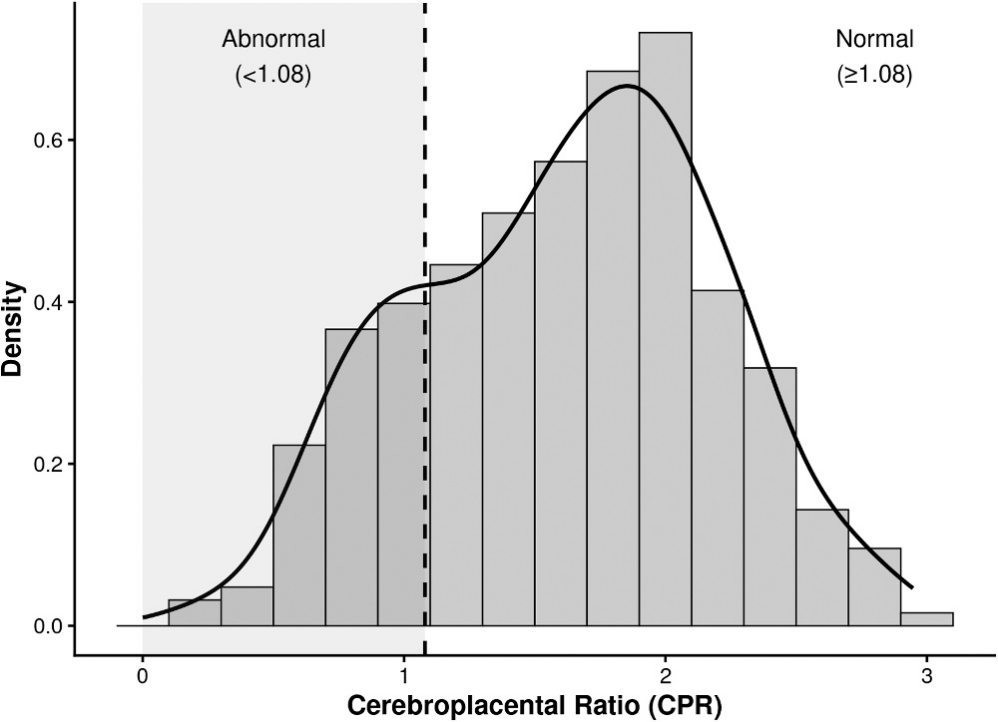
Distribution of cerebroplacental ratio values, Histogram showing the distribution of cerebroplacental ratio (CPR) values across the study population (n = 314 term pregnancies undergoing labor induction or augmentation). The vertical dashed red line indicates the pre-specified threshold of 1.08, corresponding approximately to the 5th percentile at term gestation. CPR was calculated as the ratio of middle cerebral artery pulsatility index (MCA-PI) to umbilical artery pulsatility index (UA-PI). Values below 1.08 were classified as abnormal, identifying 51 participants (16.2%; shaded in red/darker color). The distribution shows a right skew with mean CPR of 1.62 ± 0.74 (range: 0.24–5.38). Normal CPR values (≥1.08) are indicated in blue/lighter color (n = 263; 83.8%).

ROC curve analysis demonstrated moderate discriminative ability of CPR for predicting adverse outcomes. Fig. 2, Fig. 3, Fig. 4 illustrates these curves. Table 8 presents diagnostic performance at our pre-specified cut-off of 1.08. Supplementary Table S2 presents sensitivity analysis using alternative CPR cut-offs.

**Table 8:** Diagnostic Performance of Cerebroplacental Ratio for Predicting Adverse Perinatal Outcomes.

**Part A: ROC Curve Characteristics**			
**Outcome**	**AUC**	**95% CI**	**P-value**
Cesarean delivery	0.71	0.64–0.78	< 0.001
NICU admission	0.78	0.62–0.94	0.003
Composite adverse neonatal outcome	0.75	0.63–0.87	< 0.001
Small for gestational age	0.74	0.65–0.83	< 0.001
Apgar < 7 at 5 min	0.77	0.61–0.93	0.002
Neonatal resuscitation	0.76	0.60–0.92	0.002
AUC: Area under the curve; CI: Confidence interval; NICU: Neonatal intensive care unit			

**Part B: Diagnostic Performance at Pre-specified Cut-off (CPR <1.08)**				
**Outcome**	**Sensitivity % (95% CI)**	**Specificity % (95% CI)**	**PPV % (95% CI)**	**NPV % (95% CI)**
Cesarean delivery	31.9 (23.5–41.2)	92.9 (88.5–96.2)	72.5 (58.3–84.1)	70.0 (64.0–75.5)
NICU admission	62.5 (24.5–91.5)	85.3 (80.8–89.1)	9.8 (3.3–21.4)	98.9 (96.7–99.8)
Composite adverse outcome	60.0 (32.3–83.7)	85.6 (81.1–89.4)	17.6 (8.4–30.9)	97.7 (95.0–99.1)
Small for gestational age	51.3 (34.8–67.6)	88.0 (83.5–91.7)	39.2 (25.8–53.9)	92.0 (88.0–95.1)
Apgar < 7 at 5 min	62.5 (24.5–91.5)	85.3 (80.8–89.1)	9.8 (3.3–21.4)	98.9 (96.7–99.8)
Neonatal resuscitation	55.6 (21.2–86.3)	85.2 (80.7–89.1)	9.8 (3.3–21.4)	98.5 (96.2–99.6)
PPV: Positive predictive value; NPV: Negative predictive value; CI: Confidence interval				

**Part C: Likelihood Ratios and Youden Index at Pre-specified Cut-off (CPR <1.08)**				
**Outcome**	**+LR (95% CI)**	**−LR (95% CI)**	**Youden Index**	**DOR (95% CI)**
Cesarean delivery	4.20 (2.56–6.89)	0.74 (0.65–0.84)	0.24	5.68 (3.04–10.62)
NICU admission	4.25 (2.04–8.86)	0.44 (0.16–1.21)	0.48	9.65 (2.14–43.51)
Composite adverse outcome	4.17 (2.21–7.87)	0.47 (0.24–0.91)	0.46	8.87 (3.04–25.89)
Small for gestational age	4.28 (2.62–6.99)	0.55 (0.40–0.77)	0.39	7.78 (3.78–16.01)
Apgar < 7 at 5 min	4.25 (2.04–8.86)	0.44 (0.16–1.21)	0.48	9.65 (2.14–43.51)
Neonatal resuscitation	3.76 (1.72–8.22)	0.52 (0.23–1.17)	0.41	7.23 (1.82–28.73)
+LR: Positive likelihood ratio; −LR: Negative likelihood ratio; DOR: Diagnostic odds ratio; CI: Confidence interval Note: The cut-off of 1.08 was pre-specified based on prior validation studies [15], [24], not derived from this dataset.				

### 3.8 Associations between individual doppler parameters and outcomes

Table 9 presents associations between individual Doppler indices and perinatal outcomes.

**Table 9:** Associations Between Doppler Parameters and Outcomes (P-value for Association).

**Outcome Variable**	**UA-PI**	**UA-RI**	**MCA-PI**	**MCA-RI**	**PSV**	**CPR**
Birth weight	0.004	< 0.001	0.007	0.281	0.684	< 0.001
Cord pH	0.106	0.092	0.081	0.068	0.465	0.007
Base excess	0.085	0.103	0.104	0.044	0.472	< 0.001
SGA	< 0.001	< 0.001	0.785	0.354	0.676	< 0.001
NICU admission	0.002	< 0.001	0.144	0.343	0.157	0.001
Cesarean delivery	0.063	0.179	0.008	0.155	0.010	< 0.001
Resuscitation	< 0.001	< 0.001	0.081	0.198	0.067	0.003
Apgar < 7	0.002	< 0.001	0.117	0.270	0.502	0.001

Bold values indicate statistical significance (P < 0.05) P-values derived from Chi-square test (categorical outcomes) or independent *t*-test/Mann-Whitney *U* test (continuous outcomes).

CPR exhibited significant associations across all outcome categories. It represented the most comprehensive marker with associations across multiple outcome domains.

### 3.9 Subgroup analysis by labor onset type

Because 60.5% of participants presented with spontaneous labor requiring augmentation rather than true elective induction, we performed subgroup analysis. We assessed whether the association between CPR and adverse outcomes differed between these groups. Table 10, Table 11 presents these results.

**Table 10:** Association Between Abnormal CPR and Cesarean Delivery by Labor Onset Type.

**Subgroup**	**n**	**Normal CPR** **Cesarean Rate (%)**	**Abnormal CPR** **Cesarean Rate (%)**	**OR**	**95% CI**	**P-value**
True induction	124	35.1	77.3	6.42	2.48–16.62	< 0.001
Augmentation	190	26.8	68.4	5.87	2.51–13.72	< 0.001
Overall	314	30.0	72.5	6.12	3.18–11.78	< 0.001
Interaction P = 0.82						

The association between abnormal CPR and cesarean delivery was consistent across both subgroups (interaction P = 0.82). This suggests that CPR associations apply regardless of labor onset type.

**Table 11:** Sensitivity Analysis: Association Between CPR and Cesarean Delivery by Indication Type.

**Indication Category**	**Normal CPR** **Cesarean Rate**	**Abnormal CPR** **Cesarean Rate**	**OR (95% CI)**	**P-value**
All cesarean indications	79/263 (30.0%)	37/51 (72.5%)	6.12 (3.18–11.78)	< 0.001
Objective indications only*	35/263 (13.3%)	21/51 (41.2%)	4.56 (2.34–8.89)	< 0.001
(FTP + failed induction)				
Subjective indication	38/263 (14.4%)	14/51 (27.5%)	2.25 (1.10–4.61)	0.027
(Non-reassuring FHR only)				

* Failure to progress (FTP) and failed induction FHR: Fetal heart rate

### 3.10 Sensitivity analysis: cesarean delivery by indication type

To assess the potential impact of clinician knowledge of CPR status on cesarean decision-making, we performed a sensitivity analysis restricting the outcome to objective cesarean indications (failure to progress and failed induction), excluding cases performed for non-reassuring fetal heart rate patterns.

Among the 116 cesarean deliveries, 56 (48.3%) were performed for objective indications: failure to progress (n = 38) and failed induction (n = 18). The remaining 52 (44.8%) were performed for non-reassuring fetal heart rate patterns, and 8 (6.9%) for other indications.

When restricting to objective indications only, the association between abnormal CPR and cesarean delivery remained statistically significant but was attenuated (OR=4.56; 95% CI: 2.34–8.89) compared to the overall analysis (OR=6.12). For cesareans performed solely for non-reassuring fetal heart rate patterns, the association was weaker (OR=2.25; 95% CI: 1.10–4.61), though still statistically significant. This pattern suggests that while a genuine biological association exists between CPR and cesarean delivery, the observed relationship may be partially amplified by clinician awareness of CPR status influencing the threshold for intervention in cases of ambiguous fetal heart rate patterns.

### 3.11 Indication for induction/augmentation by CPR status

Women with abnormal CPR were significantly more likely to have high-risk indications for intervention compared to those with normal CPR. Table 12 presents the distribution of indications stratified by CPR status.

**Table 12:** Distribution of CPR Status by Indication for Induction/Augmentation.

**Indication Category**	**Normal CPR n = 263 (%)**	**Abnormal CPR n = 51 (%)**	P-value*
Spontaneous labor (augmentation)	162 (61.6)	28 (54.9)	0.378
High-risk indications:			
Suspected fetal compromise	25 (9.5)	9 (17.6)	0.087
Suspected IUGR	5 (1.9)	3 (5.9)	0.118
Preeclampsia	5 (1.9)	3 (5.9)	0.118
Oligohydramnios	4 (1.5)	3 (5.9)	0.066
Combined high-risk indications†	39 (14.8)	18 (35.3)	0.001
Low-risk indications:			
Post-term pregnancy	36 (13.7)	7 (13.7)	0.994
Maternal request/other	26 (9.9)	8 (15.7)	0.226

* Chi-square test Includes suspected fetal compromise, IUGR, preeclampsia, oligohydramnios

Women with abnormal CPR were significantly more likely to have high-risk indications for delivery (35.3% vs 14.8%; P = 0.001). Specifically, suspected fetal compromise was numerically more common in the abnormal CPR group (17.6% vs 9.5%; P = 0.087), though this did not reach statistical significance. This suggests potential residual confounding, as the clinical conditions prompting CPR assessment may independently predict adverse outcomes.

## 4 Discussion

### 4.1 Principal findings

This study of 314 term pregnancies undergoing labor induction or augmentation demonstrated that low CPR (<1.08) was significantly associated with multiple adverse perinatal outcomes. Fetuses with abnormal CPR showed six-fold increased odds of cesarean delivery (adjusted OR=6.12; 95% CI: 3.18–11.78), though sensitivity analysis restricted to objective cesarean indications showed attenuation of this association (OR=4.56), suggesting the primary relationship may be partially influenced by clinician awareness of CPR status**.** They showed nearly ten-fold increased odds of NICU admission (adjusted OR=9.54; 95% CI: 2.11–43.12), though this estimate carries substantial uncertainty due to the small number of NICU admissions (n = 8). They also showed over seven-fold increased odds of composite adverse neonatal outcome (adjusted OR=7.28; 95% CI: 2.68–19.78). CPR demonstrated associations across a broader range of outcome domains compared to individual Doppler indices, supporting its utility as an integrative hemodynamic marker**.**

The prevalence of abnormal CPR in our population (16.2%) aligns with comparable settings. Jamal et al. [27] documented 15.6% prevalence in term appropriate-for-gestational-age pregnancies. The meta-analysis by Vollgraff Heidweiller-Schreurs et al. [14] reported 18% across pooled studies. This consistency suggests that approximately one in six term pregnancies exhibits hemodynamic changes detectable through CPR assessment.

### 4.2 Comparison with published literature

Our findings regarding the association between low CPR and operative delivery accord with previous investigations. Mecke et al. [15] reported a threefold increase in cesarean delivery for fetal distress **in** appropriate-for-gestational-age fetuses with CPR < 1.08 in their German cohort. Anand et al. [28] documented nearly ten-fold increased cesarean odds in uncomplicated term pregnancies with CPR < 1.0 (OR: 9.65; 95% CI: 3.46–26.93). Our observed OR of 6.12 falls within this range, supporting the robustness of the CPR-cesarean association across populations.

The higher OR reported by Anand et al. [28] compared to our findings may reflect several methodological differences. First, they used a lower cut-off (CPR <1.0 vs. our 1.08), which would select a more severely affected subgroup. Second, their study included only uncomplicated pregnancies, whereas our cohort included some women with complications such as preeclampsia and oligohydramnios. Third, population-specific factors may influence the CPR-outcome relationship.

The association between abnormal CPR and neonatal acidemia corroborates earlier work by Morales-Rosello et al. [23], who demonstrated significant correlations between low CPR and reduced umbilical arterial pH values. Our finding of lower cord arterial pH (7.17 vs. 7.20; P = 0.007) extends these observations to the pre-induction and augmentation setting. We also found more negative base excess (−6.62 vs. −6.11 mmol/L; P < 0.001) in the abnormal CPR group. While absolute differences were modest and mean values remained within normal ranges, these findings are clinically relevant, suggesting that CPR may identify fetuses with reduced metabolic reserve that are vulnerable to intrapartum stress.

Regarding NICU admission, our results align with Golshahi et al. [29], who found significant associations between low CPR and NICU admission in low-risk term pregnancies. The wide confidence interval for our NICU admission OR (95% CI: 2.11–43.12) reflects the small number of NICU admissions (n = 8) in our cohort and indicates substantial uncertainty in the point estimate. This represents a limitation of our study. Larger multicenter studies would be required to estimate this association with greater precision. Ho et al. [19] noted that birthweight may independently influence the relationship between CPR and NICU admission. Our multivariable analysis partially supports this finding, as SGA was an independent predictor of composite adverse outcome.

The meta-analysis by Conde-Agudelo et al. [13] synthesized 18 studies (>55,000 pregnancies) and found that low CPR significantly predicted composite adverse perinatal outcome (OR 2.3, 95% CI: 1.7–3.2) and emergency cesarean for fetal distress (OR 2.5, 95% CI: 1.9–3.4) in suspected FGR. However, predictive accuracy was substantially lower in unselected or low-risk populations (pooled AUC 0.67 for adverse outcome), consistent with our findings (AUC 0.71–0.78). This supports the principle that CPR performs best in enriched populations where pre-test probability of placental insufficiency is higher.

The systematic review by Akolekar et al. [20] at 35–37 weeks in 16,000 + pregnancies found that CPR < 5th percentile predicted stillbirth (aOR 5.2) and neonatal unit admission (aOR 2.4), but had limited ability to predict cesarean for fetal distress in unselected populations (detection rate 15% at 10% false positive rate). These findings parallel our observation of modest sensitivity (32%) but high specificity (93%) for cesarean delivery.

Taken together, the evidence suggests CPR has established biological validity but limited population-level screening performance, particularly for subjective outcomes like cesarean delivery.

An important observation was the differential pattern of associations of individual Doppler parameters versus CPR. UA-PI and UA-RI demonstrated significant associations with growth-related outcomes and acute perinatal compromise, but did not predict cesarean delivery or acid-base disturbances. Conversely, MCA-PI was associated with cesarean delivery but not most neonatal outcomes. CPR, by integrating both vascular beds, exhibited associations across the broadest range of outcomes. This finding is consistent with the meta-analysis by Vollgraff Heidweiller-Schreurs et al. [14], who concluded that CPR may offer advantages when considering multiple outcome domains simultaneously.

### 4.3 Pathophysiological considerations

The observed associations reflect fetal hemodynamic adaptation to placental insufficiency. When placental function becomes compromised, the fetus redistributes cardiac output, prioritizing perfusion of vital organs, particularly the brain [11]. This "brain-sparing" effect manifests as decreased cerebrovascular resistance, producing lower MCA-PI values. It may be accompanied by increased placental vascular resistance, producing higher UA-PI values. Together, these changes result in reduced CPR.

Brain-sparing maintains cerebral oxygen delivery under relative hypoxemia, but indicates that the fetus operates near compensatory limits. When additional stressors are imposed, such as the repetitive reductions in uteroplacental perfusion caused by uterine contractions, these fetuses may decompensate more readily, manifesting as intrapartum fetal distress [21].

The association between abnormal CPR and neonatal acidemia has important implications, suggesting that some metabolic compromise may already be present at the time of pre**-**labor assessment. Although base excess values in our abnormal CPR group remained within conventionally reassuring ranges (>−12 mmol/L), the significantly more negative values are noteworthy, potentially indicating a trajectory toward metabolic acidosis during labor.

### 4.4 Clinical implications

Our findings have several clinical implications. First, the 16.2% prevalence of abnormal CPR among term pregnancies undergoing induction or augmentation is substantial, suggesting that a meaningful proportion of fetuses may have unrecognized vulnerability to labor stress. However, the moderate discriminative ability (AUC 0.71–0.78) and low sensitivity (32–63%) indicate that CPR has limited value as a standalone screening test. Many fetuses destined for adverse outcomes will have normal CPR values, and a normal CPR should not provide false reassurance. Routine pre**-**labor CPR assessment could potentially identify some of these fetuses, enabling enhanced intrapartum monitoring, modified induction protocols, or alternative delivery planning, though prospective trials are needed to establish whether such CPR-guided management improves outcomes.

Second, the broader pattern of associations demonstrated by CPR compared to individual Doppler indices is relevant, supporting the use of CPR as the preferred single parameter when only one measurement is feasible.

Third, the significant associations with acid-base parameters and neonatal resuscitation requirements are noteworthy, suggesting CPR may help identify deliveries requiring neonatal resuscitation preparedness.

However, several important caveats apply. CPR should not serve as a sole determinant of delivery mode or timing. Rather, abnormal CPR should prompt integration with other clinical factors, including indications for induction, cervical status, fetal biometry, and maternal conditions. The high specificity (85–92%) but modest sensitivity (32–63%) of the 1.08 cut-off means that while most women with abnormal CPR will experience adverse outcomes, many adverse outcomes will occur in women with normal CPR.

### 4.5 Strengths and limitations

This study has several methodological strengths. The sample size (n = 314) provided adequate statistical power for the primary outcome. The single-center design with standardized protocols minimized measurement variability. Comprehensive outcome ascertainment was achieved, including objective measures (cord blood gases) alongside clinical outcomes, enabling a nuanced characterization of perinatal status. Multivariable analyses adjusting for important confounders strengthen the inference of independent associations.

This study also has important limitations that warrant careful consideration:

First and most importantly, clinicians managing labor were not blinded to Doppler results. This represents the most significant potential source of bias in our study, particularly for the cesarean delivery outcome. Clinicians aware of abnormal CPR values may have had a lower threshold for proceeding to cesarean delivery when faced with ambiguous fetal heart rate patterns. This could substantially inflate the observed association between CPR and cesarean delivery. The magnitude of this bias is difficult to quantify. To illustrate: if physicians were twice as likely to perform cesarean for borderline fetal heart rate patterns when CPR was abnormal, the true OR would be considerably lower than our observed value of 6.12.

Our sensitivity analysis restricted to objective cesarean indications (failure to progress and failed induction) showed attenuation of the association (OR=4.56 vs. 6.12 for all indications), suggesting that observer bias may account for part, but not all, of the observed relationship. The association for cesareans performed solely for non-reassuring fetal heart rate patterns was weaker (OR=2.25), supporting the interpretation that clinician knowledge of CPR status may have lowered the threshold for intervention. Therefore, the cesarean delivery finding should be interpreted with considerable caution, and well-designed prospective studies with clinician blinding are essential to establish the true predictive value of CPR for this outcome. Importantly, this bias is less likely to affect objective neonatal outcomes (cord pH, Apgar scores, NICU admission), which were determined independently of clinician knowledge about CPR status.

Second, our cross-sectional design establishes an association but cannot demonstrate causation. The observed relationships between CPR and adverse outcomes may be confounded by unmeasured variables. Prospective interventional studies with randomization to CPR-guided versus standard management would be required to establish causal relationships and demonstrate clinical utility.

Third, 60.5% of participants presented with spontaneous labor requiring augmentation rather than true elective induction. Although our subgroup analysis demonstrated similar associations in both groups (interaction P = 0.82), the physiological context of augmentation differs from induction. Women requiring augmentation have already demonstrated the capacity to initiate labor spontaneously, whereas those undergoing induction have not. Our results may not fully generalize to purely elective induction populations.

Fourth, the relatively low incidence of serious adverse outcomes resulted in wide confidence intervals for some analyses. NICU admission occurred in only 8 cases (2.5%), yielding a NICU admission OR of 9.54 with a 95% CI of 2.11–43.12. This wide interval indicates considerable uncertainty in the point estimate. Larger multicenter studies with at least 1000–2000 participants would be needed to obtain precise estimates of associations with rare outcomes such as NICU admission, low Apgar scores, and neonatal resuscitation.

Fifth, we employed a fixed CPR cut-off (1.08) rather than gestational age-specific percentiles. This approach aligns with recent evidence supporting fixed thresholds at term [15], [24]. However, optimal cut-off determination requires larger population-based studies with external validation.

Sixth, all Doppler assessments were performed by a single experienced sonographer, ensuring measurement consistency but limiting generalizability to settings with different operator expertise and ultrasound equipment. Reproducibility studies with multiple operators would strengthen confidence in clinical applicability.

Seventh, women with abnormal CPR were more than twice as likely to have high-risk indications for delivery (35.3% vs 14.8%). Although our multivariable models adjusted for several confounders, we cannot exclude residual confounding by the underlying clinical conditions that prompted delivery. For example, women with preeclampsia may have adverse outcomes related to the maternal condition itself, independent of CPR status.

Eighth, Bishop score was not systematically recorded at the time of Doppler assessment. Cervical favorability strongly predicts cesarean delivery in the induction setting, and its omission from our multivariable models may have resulted in residual confounding of the CPR-cesarean association. Future studies should include Bishop score as a covariate to isolate the independent contribution of CPR to cesarean risk.

Ninth, the mean cord arterial pH (7.199) was at the lower boundary of normal, which may reflect our population enriched for risk factors (16% with suspected fetal compromise or IUGR as indication). However, median pH was 7.21 and only 3.8% had pH < 7.10, indicating that most neonates were not significantly acidemic.

Finally, we did not assess long-term neurodevelopmental outcomes. The ultimate goal of antepartum surveillance is optimization of long-term child outcomes, and short-term markers such as Apgar scores and cord pH may not perfectly predict long-term neurodevelopment.

### 4.6 Future research directions

Several research directions emerge from our findings:

Blinded prospective studies: The most pressing need is for prospective studies in which clinicians managing labor are blinded to CPR results. This would eliminate the potential bias affecting cesarean delivery outcomes and provide unbiased estimates of CPR's predictive value.

Large multicenter cohorts: Studies enrolling 1000–2000 or more participants across multiple centers would provide precise estimates for rare outcomes and enable robust subgroup analyses.

Randomized controlled trials: Ultimately, randomized trials comparing CPR-guided management versus standard care are needed. Such studies should incorporate standardized management algorithms with defined responses to abnormal CPR values and assess both benefits (reduced adverse neonatal outcomes) and potential harms (increased cesarean rates without neonatal benefit).

Long-term follow-up: Studies incorporating neurodevelopmental assessment at 2–5 years would establish whether CPR-detected fetal compromise translates into meaningful long-term differences.

Integration with other markers: Research examining CPR in combination with computerized cardiotocography, amniotic fluid assessment, and placental function biomarkers may improve predictive accuracy.

Machine learning approaches: Advanced analytical methods incorporating multiple variables could optimize risk stratification, though such models require rigorous external validation before clinical implementation.

## 5 Conclusions

In this study of 314 term pregnancies undergoing labor induction or augmentation, low CPR (<1.08) was independently associated with adverse perinatal outcomes, including cesarean delivery, NICU admission, neonatal acidemia, and low Apgar scores. However, the strong association with cesarean delivery (aOR=6.12) must be interpreted with substantial caution: clinicians were not blinded to Doppler results, and this may have created a lower threshold for cesarean delivery when CPR was abnormal, thereby artificially inflating the observed association. Our sensitivity analysis restricted to objective cesarean indications showed attenuation of this relationship (OR=4.56), suggesting that part, but not all, of the observed association may reflect observer bias. The associations with objective outcomes (cord pH, NICU admission) are less susceptible to this bias and suggest genuine hemodynamic compromise in some fetuses with low CPR.CPR demonstrated associations across a broader range of outcome domains compared to individual Doppler indices, supporting its physiological validity as an integrative marker of fetal hemodynamic status.

The moderate discriminative ability (AUC 0.71–0.78) and low sensitivity (32–63%) indicate that CPR has limited value as a standalone screening test. Many fetuses destined for adverse outcomes will have normal CPR values, and CPR should not be used to guide clinical decision-making in isolation.

These findings suggest that pre-labor CPR assessment may contribute to risk stratification in term pregnancies requiring obstetric intervention to achieve delivery. CPR has moderate predictive ability for adverse outcomes and should not alone guide clinical decisions.

Findings require confirmation in more studies.well-designed prospective trials with clinician blinding and larger sample sizes are needed to establish whether CPR-guided management improves perinatal outcomes and to quantify the true predictive value of this marker independent of observer bias.

## CRediT authorship contribution statement

**Neda Rashidian:** Writing – review & editing, Writing – original draft, Methodology, Investigation, Formal analysis. **Peyman Moshari:** Writing – review & editing, Writing – original draft, Software, Methodology, Investigation, Conceptualization. **Fatemeh Bahadori:** Writing – review & editing, Validation, Supervision, Resources, Project administration, Investigation, Data curation, Conceptualization. **Leila Majdi:** Writing – review & editing, Writing – original draft, Visualization, Validation, Software, Resources, Methodology, Investigation, Formal analysis.

## Ethics approval and consent to participate

This study was approved by the Ethics Committee of Urmia University of Medical Sciences (Ethics Code: IR.UMSU.REC.1401.159). All participants provided written informed consent.

## Consent for publication

Not applicable.

## Funding

No external funding was received.

## Declaration of Competing Interest

The authors declare that they have no known competing financial interests or personal relationships that could have appeared to influence the work reported in this paper.

## Data Availability

The datasets used and analyzed during this study are available from the corresponding author upon reasonable request.

## Footnotes

Appendix A Supplementary data associated with this article can be found in the online version at doi:10.1016/j.eurox.2026.100481.
