# Supplementary material for: Cerebroplacental ratio and middle cerebral artery doppler indices as associations with maternal and neonatal outcomes in term pregnancies undergoing labor induction or augmentation: A cross-sectional study
Source: Eur J Obstet Gynecol Reprod Biol X. 2026 Jul 31;31:100481. doi: 10.1016/j.eurox.2026.100481 (PMC13471314; doi:10.1016/j.eurox.2026.100481)
Supplement: Supplementary file 1 [file mmc1.docx]

## SUPPLEMENTARY MATERIALS

## ****Supplementary Table S1. Complete Baseline Characteristics Stratified by CPR Status****

| **Characteristic** | **Normal CPR (n=263)** | **Abnormal CPR (n=51)** | **Test Statistic** | **P-value** |
| --- | --- | --- | --- | --- |
| **Maternal Demographics** |  |  |  |  |
| Age (years), mean ± SD | 30.30 ± 7.37 | 28.60 ± 7.74 | t=1.59 | 0.113 |
| Age categories, n (%) |  |  |  |  |
| <20 years | 8 (3.0%) | 4 (7.8%) | χ²=5.82 | 0.121 |
| 20-29 years | 128 (48.7%) | 28 (54.9%) |  |  |
| 30-39 years | 112 (42.6%) | 17 (33.3%) |  |  |
| ≥40 years | 15 (5.7%) | 2 (3.9%) |  |  |
| **Gravidity** |  |  |  |  |
| Primigravida | 89 (33.8%) | 21 (41.2%) | χ²=6.23 | 0.398 |
| Gravida 2 | 65 (24.7%) | 11 (21.6%) |  |  |
| Gravida 3 | 50 (19.0%) | 8 (15.7%) |  |  |
| Gravida 4 | 35 (13.3%) | 7 (13.7%) |  |  |
| Gravida ≥5 | 24 (9.1%) | 4 (7.8%) |  |  |
| **Parity** |  |  |  |  |
| Nulliparous | 102 (38.8%) | 24 (47.1%) | χ²=1.24 | 0.265 |
| Multiparous | 161 (61.2%) | 27 (52.9%) |  |  |
| Parity 1 | 78 (29.7%) | 14 (27.5%) |  |  |
| Parity 2 | 58 (22.1%) | 9 (17.6%) |  |  |
| Parity ≥3 | 25 (9.5%) | 4 (7.8%) |  |  |
| **Gestational Age at Assessment** |  |  |  |  |
| Gestational age (weeks), mean ± SD | 39.31 ± 1.00 | 39.18 ± 1.12 | t=0.97 | 0.334 |
| 37 weeks | 19 (7.2%) | 5 (9.8%) | χ²=3.45 | 0.486 |
| 38 weeks | 51 (19.4%) | 11 (21.6%) |  |  |
| 39 weeks | 83 (31.6%) | 15 (29.4%) |  |  |
| 40 weeks | 96 (36.5%) | 16 (31.4%) |  |  |
| 41 weeks | 14 (5.3%) | 4 (7.8%) |  |  |
| **Body Mass Index (BMI)** |  |  |  |  |
| Pre-pregnancy BMI (kg/m²), mean ± SD | 24.8 ± 3.6 | 25.4 ± 4.1 | t=1.12 | 0.264 |
| BMI <18.5 (underweight) | 12 (4.6%) | 3 (5.9%) | χ²=2.15 | 0.542 |
| BMI 18.5-24.9 (normal) | 142 (54.0%) | 24 (47.1%) |  |  |
| BMI 25-29.9 (overweight) | 86 (32.7%) | 18 (35.3%) |  |  |
| BMI ≥30 (obese) | 23 (8.7%) | 6 (11.8%) |  |  |
| **Medical History** |  |  |  |  |
| Chronic hypertension | 8 (3.0%) | 3 (5.9%) | Fisher's | 0.398 |
| Pre-gestational diabetes | 6 (2.3%) | 2 (3.9%) | Fisher's | 0.618 |
| Thyroid disorder | 15 (5.7%) | 4 (7.8%) | Fisher's | 0.531 |
| Previous cesarean delivery (in multiparous) | 68/161 (42.2%) | 14/27 (51.9%) | χ²=0.85 | 0.357 |
| **Smoking Status** |  |  |  |  |
| Never smoker | 238 (90.5%) | 45 (88.2%) | χ²=0.28 | 0.869 |
| Former smoker | 18 (6.8%) | 4 (7.8%) |  |  |
| Current smoker | 7 (2.7%) | 2 (3.9%) |  |  |
| **Consanguinity** |  |  |  |  |
| Consanguineous marriage | 78 (29.7%) | 17 (33.3%) | χ²=0.28 | 0.595 |

SD: Standard deviation

**Supplementary Table S2. Sensitivity Analysis of Predictive Performance Using Alternative CPR Cut-offs**

### **Part A: Alternative Cut-off 1.0 (10th percentile approximation)**

| **Outcome** | **Prevalence of Abnormal CPR** | **Sensitivity %** | **Specificity %** | **PPV %** | **NPV %** | **AUC (95% CI)** |
| --- | --- | --- | --- | --- | --- | --- |
| Cesarean delivery | 12.1% (n=38) | 28.4 | 94.4 | 76.3 | 68.2 | 0.71 (0.64-0.78) |
| NICU admission | 12.1% (n=38) | 75.0 | 88.9 | 15.8 | 99.3 | 0.82 (0.69-0.95) |
| Composite adverse outcome | 12.1% (n=38) | 66.7 | 89.3 | 26.3 | 97.8 | 0.78 (0.66-0.90) |
| SGA | 12.1% (n=38) | 56.4 | 90.9 | 46.1 | 93.5 | 0.74 (0.65-0.83) |

### **Part B: Alternative Cut-off 5th Percentile for Gestational Age**

**5th percentile values by gestational age (derived from published references [15,24]):**

- 37-38 weeks: CPR <1.05
- 39 weeks: CPR <1.08
- 40-41 weeks: CPR <1.10

| **Outcome** | **Prevalence of Abnormal CPR** | **Sensitivity %** | **Specificity %** | **PPV %** | **NPV %** | **AUC (95% CI)** |
| --- | --- | --- | --- | --- | --- | --- |
| Cesarean delivery | 14.6% (n=46) | 30.2 | 93.4 | 74.0 | 69.8 | 0.70 (0.63-0.77) |
| NICU admission | 14.6% (n=46) | 62.5 | 86.9 | 10.9 | 98.9 | 0.75 (0.61-0.89) |
| Composite adverse outcome | 14.6% (n=46) | 60.0 | 87.2 | 19.6 | 97.6 | 0.74 (0.62-0.86) |
| SGA | 14.6% (n=46) | 53.8 | 89.1 | 43.5 | 92.5 | 0.71 (0.62-0.80) |

### **Part C: Alternative Cut-off 10th Percentile for Gestational Age**

**10th percentile values by gestational age:**

- 37-38 weeks: CPR <1.20
- 39 weeks: CPR <1.25
- 40-41 weeks: CPR <1.28

| **Outcome** | **Prevalence of Abnormal CPR** | **Sensitivity %** | **Specificity %** | **PPV %** | **NPV %** | **AUC (95% CI)** |
| --- | --- | --- | --- | --- | --- | --- |
| Cesarean delivery | 24.5% (n=77) | 44.8 | 85.4 | 60.3 | 75.7 | 0.68 (0.61-0.75) |
| NICU admission | 24.5% (n=77) | 87.5 | 77.8 | 9.1 | 99.6 | 0.83 (0.73-0.93) |
| Composite adverse outcome | 24.5% (n=77) | 80.0 | 78.5 | 15.6 | 98.7 | 0.79 (0.68-0.90) |
| SGA | 24.5% (n=77) | 69.2 | 80.0 | 35.1 | 94.5 | 0.75 (0.67-0.83) |

### **Part D: Comparison of Cut-offs**

| **Cut-off Strategy** | **Optimal for** | **Advantages** | **Disadvantages** |
| --- | --- | --- | --- |
| **CPR <1.0** | High specificity | Very high specificity (94%), few false positives | Low sensitivity (28%), misses many cases |
| **CPR <1.08 (used in study)** | Balanced performance | Balance of sensitivity/specificity, validated in multiple studies | Moderate sensitivity (32%) |
| **5th percentile (GA-specific)** | Term pregnancies | Accounts for gestational variation | Complex calculation, similar performance to 1.08 |
| **10th percentile (GA-specific)** | High sensitivity | Better sensitivity (45-87%) | Lower specificity, more false positives |

### **Interpretation:**

The pre-specified cut-off of **1.08** offers a reasonable balance between sensitivity and specificity for term pregnancies undergoing labor induction/augmentation. Lower cut-offs (1.0) increase specificity but substantially reduce sensitivity. Higher cut-offs (10th percentile) improve sensitivity for some outcomes (particularly NICU admission) but increase false positive rates. The choice of cut-off should depend on the clinical context: screening programs may favor higher sensitivity (10th percentile), whereas risk stratification for intensive monitoring may favor higher specificity (1.0 or 1.08).

PPV: Positive predictive value; NPV: Negative predictive value; AUC: Area under the curve; CI: Confidence interval; SGA: Small for gestational age; NICU: Neonatal intensive care unit; GA: Gestational age

**Supplementary Table S3. Correlation Matrix (Spearman Coefficients) for All Doppler Parameters**

|  | **MCA-PI** | **MCA-RI** | **MCA-PSV** | **UA-PI** | **UA-RI** | **CPR** | **Birth Weight** | **Cord pH** | **Base Excess** |
| --- | --- | --- | --- | --- | --- | --- | --- | --- | --- |
| **MCA-PI** | 1.000 |  |  |  |  |  |  |  |  |
| **MCA-RI** | 0.856** | 1.000 |  |  |  |  |  |  |  |
| **MCA-PSV** | -0.124* | -0.089 | 1.000 |  |  |  |  |  |  |
| **UA-PI** | -0.245** | -0.198** | 0.056 | 1.000 |  |  |  |  |  |
| **UA-RI** | -0.267** | -0.221** | 0.043 | 0.912** | 1.000 |  |  |  |  |
| **CPR** | 0.723** | 0.654** | -0.098 | -0.678** | -0.689** | 1.000 |  |  |  |
| **Birth Weight** | 0.152** | 0.061 | 0.023 | -0.162** | -0.201** | 0.245** | 1.000 |  |  |
| **Cord pH** | 0.098 | 0.103 | -0.042 | -0.091 | -0.095 | 0.151** | 0.089 | 1.000 |  |
| **Base Excess** | 0.092 | 0.114* | -0.038 | -0.096 | -0.092 | 0.203** | 0.067 | 0.685** | 1.000 |
| **Gestational Age** | -0.078 | -0.065 | 0.034 | 0.082 | 0.091 | -0.112* | 0.187** | -0.045 | -0.038 |
| **Maternal Age** | 0.034 | 0.028 | -0.012 | -0.023 | -0.019 | 0.041 | -0.098 | 0.056 | 0.047 |

* P<0.05; ** P<0.01

**Interpretation:**

**Strong positive correlations (r > 0.7):**

- MCA-PI and MCA-RI (r=0.856) — expected, as both measure MCA resistance
- UA-PI and UA-RI (r=0.912) — expected, as both measure UA resistance
- MCA-PI and CPR (r=0.723) — expected, as MCA-PI is the numerator of CPR
- Cord pH and Base Excess (r=0.685) — expected, both measure acid-base status

**Moderate negative correlations (r = -0.6 to -0.7):**

- CPR and UA-PI (r=-0.678) — expected, as UA-PI is the denominator of CPR (inversely related)
- CPR and UA-RI (r=-0.689) — similar relationship

**Weak but significant correlations:**

- CPR and birth weight (r=0.245, P<0.01) — higher CPR associated with higher birth weight
- CPR and cord pH (r=0.151, P<0.01) — higher CPR associated with better pH
- CPR and base excess (r=0.203, P<0.01) — higher CPR associated with less negative base excess

**Key clinical insights:**

1. CPR integrates information from both MCA and UA Doppler, but is not simply redundant with either individual parameter
2. The weak-to-moderate correlations between CPR and neonatal outcomes (birth weight, pH, base excess) explain the moderate discriminative ability (AUC 0.71-0.78)
3. Absence of strong correlation between Doppler parameters and maternal age or gestational age (at term) supports the use of fixed CPR cut-off rather than age-adjusted values
4. Low correlation between MCA-PSV and other parameters suggests it measures a different aspect of fetal hemodynamics (absolute flow velocity vs. resistance indices)

MCA: Middle cerebral artery; UA: Umbilical artery; PI: Pulsatility index; RI: Resistive index; PSV: Peak systolic velocity; CPR: Cerebroplacental ratio

**Supplementary File S4.** **Completed STROBE checklist for cross-sectional studies.**

| **Item No.** | **Recommendation** | **Location in Manuscript** |
| --- | --- | --- |
| **Title and Abstract** |  |  |
| 1 | (a) Indicate the study's design with a commonly used term in the title or abstract | Title, Abstract |
|  | (b) Provide in the abstract an informative and balanced summary | Abstract |
| **Introduction** |  |  |
| 2 | Explain the scientific background and rationale | Section 1, paragraphs 1-6 |
| 3 | State specific objectives, including any pre-specified hypotheses | Section 1, final paragraph |
| **Methods** |  |  |
| 4 | Present key elements of study design early in the paper | Section 2.1 |
| 5 | Describe the setting, locations, and relevant dates | Section 2.1 |
| 6 | Give the eligibility criteria, sources, and methods of selection | Section 2.2 |
| 7 | Clearly define all outcomes, exposures, predictors, potential confounders | Sections 2.4, 2.5 |
| 8 | For each variable, give sources of data and details of assessment | Section 2.4 |
| 9 | Describe any efforts to address potential sources of bias | Sections 2.5, 4.5 |
| 10 | Explain how the study size was arrived at | Section 2.3 |
| 11 | Explain how quantitative variables were handled | Section 2.6 |
| 12 | Describe all statistical methods | Section 2.6 |
| **Results** |  |  |
| 13 | Report numbers of individuals at each stage of study | Section 3.1, Figure 1 |
| 14 | Give characteristics of study participants | Section 3.1, Tables 1-2 |
| 15 | Report numbers of outcome events or summary measures | Sections 3.3-3.5 |
| 16 | Give unadjusted estimates and confounder-adjusted estimates | Tables 12-13 |
| 17 | Report other analyses done—subgroup analyses and sensitivity analyses | Sections 3.7, 3.9 |
| **Discussion** |  |  |
| 18 | Summarize key results with reference to study objectives | Section 4.1 |
| 19 | Discuss limitations, including sources of potential bias | Section 4.5 |
| 20 | Give a cautious overall interpretation of results | Sections 4.4, 5 |
| 21 | Discuss the generalizability of the results | Sections 4.4, 4.5 |
| **Other Information** |  |  |
| 22 | Give the source of funding and role of funders | Declarations |

**Supplementary Table S5. Doppler Parameters by Gestational Week**

| **Parameter** | **37 weeks (n=24)** | **38 weeks (n=62)** | **39 weeks (n=98)** | **40 weeks (n=112)** | **41 weeks (n=18)** | **Overall (n=314)** | **P-value*** |
| --- | --- | --- | --- | --- | --- | --- | --- |
| **MCA-PI** | 1.26±0.32 | 1.27±0.32 | 1.15±0.21 | 1.26±0.58 | 1.22±0.11 | 1.24±0.42 | 0.489 |
|  | (0.81-1.80) | (0.90-2.16) | (0.90-1.60) | (0.63-3.28) | (1.03-1.39) | (0.63-3.28) |  |
| **MCA-RI** | 0.70±0.18 | 0.69±0.12 | 0.69±0.09 | 0.69±0.10 | 0.68±0.08 | 0.68±0.11 | 0.703 |
|  | (0.46-1.00) | (0.42-0.90) | (0.53-0.84) | (0.46-0.90) | (0.54-0.80) | (0.42-1.00) |  |
| **UA-PI** | 0.89±0.49 | 0.84±0.23 | 0.81±0.18 | 0.79±0.37 | 0.94±0.61 | 0.83±0.36 | 0.303 |
|  | (0.53-2.41) | (0.50-1.79) | (0.53-1.20) | (0.40-2.64) | (0.52-2.08) | (0.40-2.64) |  |
| **UA-RI** | 0.55±0.17 | 0.56±0.10 | 0.55±0.08 | 0.51±0.06 | 0.67±0.41† | 0.54±0.13 | 0.017 |
|  | (0.32-1.00) | (0.38-0.83) | (0.40-0.74) | (0.36-0.65) | (0.40-1.44) | (0.32-1.44) |  |
| **MCA-PSV (cm/s)** | 46.79±16.96 | 45.93±15.31 | 51.47±17.95 | 40.30±14.86 | 43.21±13.30 | 46.07±16.09 | 0.266 |
|  | (19.10-80.0) | (17.00-79.9) | (17.00-80.0) | (18.00-75.3) | (29.80-64.0) | (17.00-80.0) |  |
| **CPR** | 1.62±0.63 | 1.63±0.56 | 1.49±0.48 | 1.77±0.95 | 1.69±0.71 | 1.62±0.74 | 0.421 |
|  | (0.75-2.73) | (0.57-2.54) | (0.91-3.02) | (0.24-5.38) | (0.56-2.67) | (0.24-5.38) |  |

Values presented as mean ± SD (range)
*Kruskal-Wallis H test
†Significantly different from 40 weeks (post-hoc P=0.016)
MCA-PI: Middle cerebral artery pulsatility index; MCA-RI: Middle cerebral artery resistive index; UA-PI: Umbilical artery pulsatility index; UA-RI: Umbilical artery resistive index; MCA-PSV: Middle cerebral artery peak systolic velocity; CPR: Cerebroplacental ratio
